# Examination of Calcium Silicate Cements with Low-Viscosity Methyl Cellulose or Hydroxypropyl Cellulose Additive

**DOI:** 10.1155/2016/4583854

**Published:** 2016-11-17

**Authors:** Toshiaki Baba, Yasuhisa Tsujimoto

**Affiliations:** ^1^Department of Endodontics, Nihon University School of Dentistry at Matsudo, Chiba, Japan; ^2^Research Institute of Oral Science, Nihon University School of Dentistry at Matsudo, Chiba, Japan

## Abstract

The purpose of this study was to improve the operability of calcium silicate cements (CSCs) such as mineral trioxide aggregate (MTA) cement. The flow, working time, and setting time of CSCs with different compositions containing low-viscosity methyl cellulose (MC) or hydroxypropyl cellulose (HPC) additive were examined according to ISO 6876-2012; calcium ion release analysis was also conducted. MTA and low-heat Portland cement (LPC) including 20% fine particle zirconium oxide (ZO group), LPC including zirconium oxide and 2 wt% low-viscosity MC (MC group), and HPC (HPC group) were tested. MC and HPC groups exhibited significantly higher flow values and setting times than other groups (*p* < 0.05). Additionally, flow values of these groups were higher than the ISO 6876-2012 reference values; furthermore, working times were over 10 min. Calcium ion release was retarded with ZO, MC, and HPC groups compared with MTA. The concentration of calcium ions was decreased by the addition of the MC or HPC group compared with the ZO group. When low-viscosity MC or HPC was added, the composition of CSCs changed, thus fulfilling the requirements for use as root canal sealer. Calcium ion release by CSCs was affected by changing the CSC composition via the addition of MC or HPC.

## 1. Introduction

Root canal fillings can seal the content of the root canal system, thereby preventing the egress of microorganisms or byproducts into periradicular tissues. An ideal root canal filling material should be biocompatible, antibacterial, nontoxic, and radiopaque and should not be resorbable or soluble in an oral environment [1]. Moreover, the material should be cost-effective, easy to handle, and closely adaptable to the cavity walls. Mineral trioxide aggregate (MTA), which is a calcium silicate cement (CSC) used in dentistry, was first developed by Torabinejad et al. [2]. MTA is used primarily for root canal filling, perforation repair, and retrofilling because MTA has unique biocompatibility [3], antibacterial properties [4], and sealability [5] and promotes hard tissue formation [6]. However, it is difficult to use because of its granular consistency, slow setting time, and initial looseness [7].

MTA contains Portland cement (PC), which comprises about 80% of the material. PC itself is generally composed of constituents such as a-lite (3CaO·SiO_2_), b-lite (2CaO·SiO_2_), aluminate (3CaO·Al_2_O_3_), ferrite (4CaO·Al_2_O_3_·Fe_2_O_3_), and gypsum (Table 1). The constituents of PC have different features; for example, b-lite has excellent long-term hardness and aluminate shows the earliest hydration. Hence, the ratio of the different cement components varies depending on the application. For example, low-heat Portland cement (LPC, Taiheiyo Cement, Japan) has a different composition from PC; for example, it is comprised of at least 40% b-lite. Compared with general PC, LPC has a low initial hydration rate, low heat of hydration, long-term hydration, and excellent long-term hardness.

**Figure 1:**
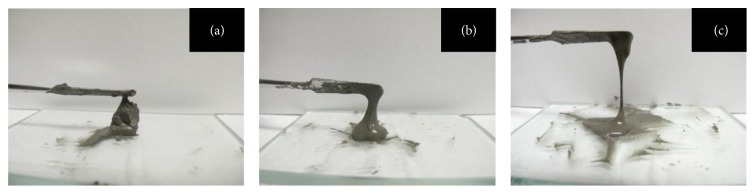
Image of the PC including 2% MC with viscosity about 4,000 (a), 400 (b), and 25 (c).

CSCs, which are represented here by MTA, contain about 20% radiopacifying agent (RA) for increased radiopacity. However, in previous reports on the use of an RA as an additive to a CSC, bismuth oxide (BO) was used and has been reported to affect various physical properties. For example, BO has been reported to reduce the compressive strength [8] and extend the setting time [9] of CSCs. Bosso-Martelo et al. [10] evaluated the physical and chemical properties of CSCs with different chemical compositions by replacing an RA with other materials. Microsized zirconium oxide (ZO) particles may be considered a potential RA for use in association with CSCs. Moreover, the addition of nanoparticulate RA affects some of CSCs' physical properties, such as decreasing their radiopacity and retarding their initial and final setting times when compared with MTA [10]. Additionally, numerous attempts have been made to improve the handling properties of MTA by adding materials to enhance viscosity [7, 11]; Ber et al. [11] reported that the addition of methyl cellulose (MC), which is cellulose ether (CE), improved the handling properties of CSCs. Furthermore, the viscosity of MC is adjustable (Figure 1). The authors reported that the addition of tantalum oxide as an RA and that of low-viscosity MC improved the flow value of CSCs such that it exceeded the reference value of the ISO standard 6876-2012: dental root sealing materials [12, 13]. Belonging to the same CE as MC, hydroxypropyl cellulose (HPC) is biocompatible and nontoxic, and its viscosity can be adjusted similar to that of MC. It is used in various applications as a medical or food additive [14]. However, to date, there have been no studies comparing the use of low-viscosity HPC as the additive in CSCs compared with using MC.

The goal of the present study is to improve the poor handling of CSCs and to develop materials that can be used as root canal sealing materials by the addition of low-viscosity MC and HPC to a CSC to replace PC with LPC and BO with ZO, which we then compare with MTA. For the purpose of this study, we examine some of the physical experiments described in ISO standard 6876-2012: dental root sealing materials [12] and experimentally determine the effects of calcium ion release.

## 2. Materials and Methods

Pro Root MTA (Dentsply Maillefer, Ballaigues, Switzerland) was used in this study alongside LPC (Taiheiyo Cement Co., Tokyo, Japan) with an addition of 20% ZO (Wako, Tokyo, Japan) with particle sizes of 10 *μ*m or less (ZO group). LPC with the addition of ZO and 2 wt% low-viscosity MC (Wako, Tokyo, Japan; referred to as MC group) or low-viscosity HPC (Nippon Soda, Tokyo, Japan; referred to as HPC group) were also examined. The viscosity and molecular weight of the CE used in this experiment are shown in Table 2.

### 2.1. Flow

Each sample was mixed, and 0.05 ± 0.005 mL of the sealer was placed on the center of a glass plate. After 3 min, another glass plate (120 ± 0.5 g) was placed on the sealer. After seven additional minutes, the major and minor diameters of the compressed material were measured. If both measurements were within 1 mm of each other, the results were recorded. The test was conducted ten times for each sample group, and the mean value was recorded. The data obtained in these flow tests were analyzed by a one-way ANOVA test for global comparison and using Tukey's test for individual comparisons; *p* values below 0.05 were considered to be statistically significant.

### 2.2. Working Time

Among the results obtained in experiment 1, the working times of groups with flow values exceeding 17 mm were measured. The same experiment was repeated at increasing time intervals between the start of mixing and the placement of the second glass plate. The working time was recorded when the disc diameter fell below 17 mm. The test was conducted five times for each sample group, and the mean value was recorded. The data obtained in the working time tests were analyzed via Student's* t*-test for individual comparisons; *p* values below 0.05 were considered to be statistically significant.

### 2.3. Setting Time

Each sample (10 mm in diameter and 1 mm high) was prepared and stored in an incubator at 37°C and 95% humidity. An indenter (100 ± 0.5 g) with a flat end (2 mm in diameter) was repeatedly lowered vertically onto the mounted sample surface. The mixing time was recorded when no indentation could be detected. The test was conducted five times for each sample group, and the mean value was recorded. The data obtained with the setting time test were analyzed by a one-way ANOVA test for global comparison and using Tukey's test for individual comparisons; *p* values below 0.05 were considered to be statistically significant.

### 2.4. Calcium Ion Release Analysis

To estimate the calcium ion release values, molds (3 mm in diameter and 2 mm high) were filled with each sample group, with one side of the mold immersed in 1000 *μ*L pure water. The analyses were performed after 3 h, 6 h, 12 h, 24 h, 3 days, 7 days, and 21 days after the mixing process by using a compact calcium ion meter B-751 (HORIBA Scientific, Tokyo, Japan). The test was conducted five times for each sample group, and the mean value was recorded. The data obtained for the concentration of calcium ions (in ppm) released after 21 days were analyzed using a one-way ANOVA test for global comparison and using Tukey's test for individual comparisons; *p* values below 0.05 were considered to be statistically significant.

## 3. Results

### 3.1. Flow

The ZO sample group exhibited a significantly higher flow value compared with the MTA group (*p* < 0.05). Both the MC and HPC groups exhibited a significantly higher flow value compared with the MTA and ZO groups (*p* < 0.05), with flow values above 17 mm, which is the reference value according to ISO 6876-2012 (Table 3).

### 3.2. Working Time

In the MC and HPC groups, a working time between 12 and 15 min was obtained (Table 3). There was no significant difference between the MC and HPC groups.

### 3.3. Setting Time

The MC and HPC sample groups had significantly retarded setting times compared to the MTA and ZO groups (*p* < 0.05), and the MC group had a significantly retarded setting time compared with the HPC group (*p* < 0.05). The setting time tests revealed that all samples had a setting time of somewhere from 30 min to 72 h, as determined by ISO 6876-2012 (Table 3).

### 3.4. Calcium Ion Release Analysis

**Figure 2:**
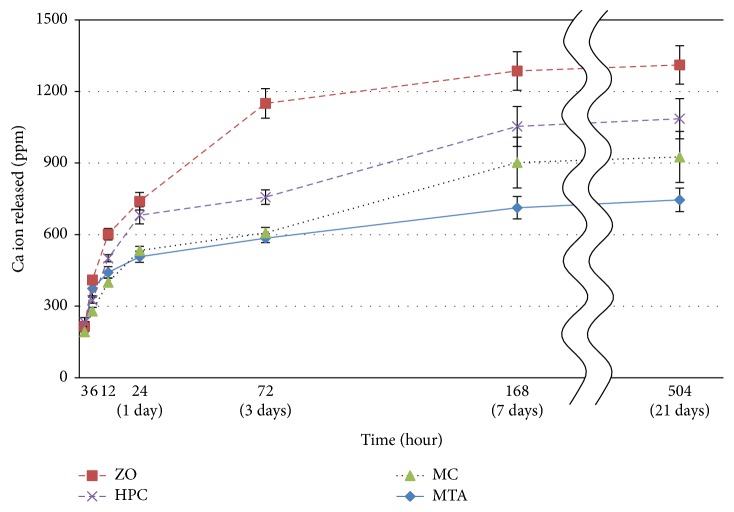
Calcium ions released after 3, 6, and 12 h as well as after 1, 3, 7, and 21 days.

The concentration of calcium ions at 21 days was as follows (the sample groups mentioned first had the highest and those mentioned last had the lowest concentration): ZO, HPC, MC, and MTA groups (Table 3). Additionally, each group displayed a value that was significantly different compared with that of any other group (*p* < 0.05). Further, the concentration of calcium ions increased rapidly within the first day for the MTA group, within 3 days for the ZO group, and within 7 days for the HPC and MC groups; after that, the increase was only moderate (Figure 2).

## 4. Discussion

The purpose of the present study was to improve the poor handling of CSCs by the addition of low-viscosity MC and HPC to a CSC to replace PC with LPC and BO with ZO, which we then compared with MTA.

MTA comprises about 80% PC and 20% BO. Another CSC was composed of about 80% LPC and 20% ZO. Compared with the regularly used PC, LPC has a slow initial hydration rate and an excellent long-term hardness. On the other hand, the addition of nanoparticulate RA affects some physical properties of the CSC when compared with MTA [10]. In the results, the ZO group exhibited a significantly higher flow value compared with the MTA group (*p* < 0.05). Moreover, with regards to the concentration of calcium ions at 21 days, the ZO group exhibited a significantly increased value compared with the MTA group (*p* < 0.05). Further, the concentration of calcium ions increased rapidly up to the first day in the MTA group and 3 days in the ZO group, after which the increase was only moderate. This result may be due to the retarded initial hydration by using LPC and using fine-grained RA particles in the ZO group. Although there was no significant difference between the ZO group and the MTA group with regards to the setting time test using the conditions laid out in ISO 6876-2012, hydration retardation seemed to have occurred. Probably, the setting time would be retarded if the experiment were carried out using another method such as the one laid out in the American Society for Testing and Materials standard. This is a subject for future analysis. In the ZO groups, there were no samples that exhibited a flow value higher than the reference value, as determined by ISO 6876-2012. Therefore, only changing the CSC composition appeared to be insufficient to allow CSC to be used as a dental root sealing material.

MC and HPC are the same type of CE. The viscosity of PC is improved by addition of CE. In this study, the MC and HPC sample groups exhibited significantly higher flow values, with values of 20 mm or over, compared with the MTA and ZO groups (*p* < 0.05); additionally, the flow values of these sample groups were higher than the 17 mm reference value given by ISO 6876-2012 (Table 3). We considered whether the result of the flow test was due to an improvement in CSC's granular consistency and the initial looseness caused by the addition of the low-viscosity CE. Moreover, working times between 12 and 15 min for the MC and HPC groups were obtained (Table 3). There is no reference value for the working time test in ISO 6876-2012. However, the working time of the MC and HPC sample groups in the present study was not good enough because other sealers have working times greater than 20 min (e.g., zinc oxide eugenol cement). In the setting time test, MC and HPC groups showed significantly increased setting times compared with the MTA and ZO groups (*p* < 0.05); further, the MC group showed a significantly increased setting time compared with the HPC group (*p* < 0.05). CE usually prolongs the setting time of PC and retards cement hydration. This occurs owing to the adsorption of CE on the hydration products of PC, such as silicate oxides and metal oxides. CE prevents hydration by covering the CSC grain [15]. This causes the high CE adsorption capacity on hydrated phases such as C-S-H and calcium hydroxide [16]. Also, this is what causes the high CE adsorption capacity on aluminate in relation to the initial hydration. Hydration retardation might be inhibited by preferential adsorption to the aluminate. It is reported that, among the same types of CE, the lower the molecular weight, the stronger the retardation effect [17]. In this study, we investigated an LPC that has a low rate and contains aluminate. Thus, we decided that the hydration retardation effect of the CE was not inhibited. In fact, the results of the calcium ion release analysis showed that the concentration of calcium ions was increased rapidly up to 3 days in the ZO group and 7 days in the HPC and MC groups, after which the increase was only moderate (Figure 2). It was suggested that this retardation difference was caused by CE, which inhibited CSC hydration. Further, it has been suggested that the ZO group increases the concentration of calcium ions at 21 days compared with the MC and HPC groups (*p* < 0.05). It was also supposed that this result occurred by the adsorption of CE on the initial hydration products of CSC. The setting time test in this study revealed that all samples had setting times of 30 min to 72 h, as determined according to ISO 6876-2012 (Table 3). However, Hsieh et al. [7] reported that the slow setting time of CSC caused it to be difficult to use. In this study, the setting time of the MC group was significantly extended, which decreased the concentration of calcium ions at 21 days compared with the ZO group (*p* < 0.05). It was considered whether this was caused by the difference in the molecular weight between MC and HPC. Therefore, we analyzed the data with different molecular weights of MC and HPC with this hypothesis in mind. For MC, which has a lower molecular weight compared with HPC, there was stronger retardation along with CE adsorption on the hydration products of CSC. We propose that, to avoid retardation of the setting time by CE addition, it is necessary to reduce the CE adsorption for the hydration product by using additives such as calcium chloride and to select high-molecular-weight CE with the same viscosity.

## 5. Conclusions

We added a low-viscosity CE to a CSC to replace PC with LPC and BO with ZO, which we then compared with MTA. We obtained materials with physical properties that fulfil some of the requirements for use as a dental root sealing material. Additionally, there was an increase in the concentration of calcium ions retarded when replacing PC with LPC and BO with ZO and further retardation with the addition of CE. The addition of CE in CSC decreased the concentration of calcium ions released.

## Figures and Tables

**Table 1:** Details of the composition of ordinary PC.

Component of ordinary PC	Characteristic
a-lite, C_3_S (about 65%) 3CaO·SiO_2_	Hydration rate: *fast.* Heat of hydration: *middle.* Initial strength: *excellent.* Long-term strength: *excellent.* Chemical resistance: *middle.* Drying shrinkage: *middle.*

b-lite, C_2_S (about 15%) 2CaO·SiO_2_	Hydration rate: *slow.* Heat of hydration: *low.* Initial strength: *poor.* Long-term strength: *excellent.* Chemical resistance: *high.* Drying shrinkage: *low.*

Aluminate, C_3_A (about 7%) 3CaO·Al_2_O_3_	Hydration rate: *fastest.* Heat of hydration: *highest.* Initial strength: *excellent.* Long-term strength: *poor.* Chemical resistance: *low.* Drying shrinkage: *high.*

Ferrite, C_4_AF (about 8%) 4CaO·Al_2_O_3_·Fe_2_O_3_	Hydration rate: *middle.* Heat of hydration: *middle.* Initial strength: *middle.* Long-term strength: *middle.* Chemical resistance: *middle.* Drying shrinkage: *middle.*

Gypsum CaSO_4_·2H_2_O	Gypsum slows down the aluminate hydration. Without addition of gypsum, PC reaction proceeds rapidly, and physical properties become poor.

**Table 2:** Properties of the CE used in this study.

	Viscosity (2%, 20°C)	Weight-average molecular weight
Methyl cellulose (MC)	20~30 mPa·s	40,000
Hydroxypropyl cellulose (HPC)	5~10 mPa·s	140,000

**Table 3:** Results of this study. Different letters indicate statistically significant differences in each test (Tukey's test, *p* < 0.05). For example, there are significant differences between a, b, and c. And, there are no significant differences between MC and HPC in flow test.

		MTA	ZO	MC	HPC	Reference value of ISO standard 6876-2012
Flow (mm) *n* = 10	Mean ± SD	9.6 ± 0.8^a^	12.2 ± 0.9^b^	22.8 ± 1.5^c^	22.7 ± 1.0^c^	16.00 mm
	Median (MIN~MAX)	9.9 (8.1~10.2)	12.0 (11.4~13.9)	22.7 (20.6~24.7)	22.9 (20.8~24.1)	

Working time (min) *n* = 5	Mean ± SD	—	—	13 ± 1^d^	14 ± 1^d^	—
	Median (MIN~MAX)	—	—	13 (12~13)	14 (12~15)	

Setting time (min) *n* = 5	Mean ± SD	31 ± 4^e^	30 ± 5^e^	118 ± 8^f^	98 ± 4^g^	30 min~72 hours
	Median (MIN~MAX)	30 (25~35)	30 (25~35)	120 (110~125)	95 (95~105)	

Ca ion released (ppm) *n* = 5	Mean ± SD	745 ± 49^h^	1311 ± 80^i^	926 ± 107^j^	1086 ± 84^k^	—
	Median (MIN~MAX)	746 (668~789)	1342 (1214~1411)	958 (799~1030)	1042 (1016~1189)	

## References

[1] Lotfi M., Ghasemi N., Rahimi S., Vosoughhosseini S., Saghiri M. A., Shahidi A. (2013). Resilon: a comprehensive literature review. *Journal of Dental Research, Dental Clinics, Dental Prospects*.

[2] Torabinejad M., Watson T. F., Ford T. R. P. (1993). Sealing ability of a mineral trioxide aggregate when used as a root end filling material. *Journal of Endodontics*.

[3] Ribeiro D. A., Matsumoto M. A., Duarte M. A. H., Marques M. E. A., Salvadori D. M. F. (2006). Ex vivo biocompatibility tests of regular and white forms of mineral trioxide aggregate. *International Endodontic Journal*.

[4] Asnaashari M., Asgary S., Khatami A. (2006). Bacterial leakage of mineral trioxide aggregates and portland cement. *Iranian Endodontic Journal*.

[5] Mohammadi Z., Khademi A. (2007). An evaluation of the sealing ability of MTA and resilon: a bacterial leakage study. *Iranian Endodontic Journal*.

[6] Shayegan A., Petein M., Abbeele A. V. (2008). Beta-tricalcium phosphate, white mineral trioxide aggregate, white Portland cement, ferric sulfate, and formocresol used as pulpotomy agents in primary pig teeth. *Oral Surgery, Oral Medicine, Oral Pathology, Oral Radiology and Endodontology*.

[7] Hsieh S.-C., Teng N.-C., Lin Y.-C. (2009). A novel accelerator for improving the handling properties of dental filling materials. *Journal of Endodontics*.

[8] Antonijevic D., Medigovic I., Zrilic M., Jokic B., Vukovic Z., Todorovic L. (2014). The influence of different radiopacifying agents on the radiopacity, compressive strength, setting time, and porosity of Portland cement. *Clinical Oral Investigations*.

[9] Grazziotin-Soares R., Nekoofar M. H., Davies T. E. (2014). Effect of bismuth oxide on white mineral trioxide aggregate: chemical characterization and physical properties. *International Endodontic Journal*.

[10] Bosso-Martelo R., Guerreiro-Tanomaru J. M., Viapiana R., Berbert F. L. C., Duarte M. A. H., Tanomaru-Filho M. (2016). Physicochemical properties of calcium silicate cements associated with microparticulate and nanoparticulate radiopacifiers. *Clinical Oral Investigations*.

[11] Ber B. S., Hatton J. F., Stewart G. P. (2007). Chemical modification of proroot MTA to improve handling characteristics and decrease setting time. *Journal of Endodontics*.

[12] International Organization for Standardization (1986). Specification for dental root canal sealing materials. *ISO*.

[13] Baba T., Izawa M., Tsujimoto Y. (2014). Physical properties of calcium silicate cement was influenced by radiopacity agent and/or anti-washout admixture. *International Journal of Microdentistry*.

[14] Sarode A., Wang P., Cote C., Worthen D. R. (2013). Low-viscosity hydroxypropylcellulose (HPC) grades SL and SSL: versatile pharmaceutical polymers for dissolution enhancement, controlled release, and pharmaceutical processing. *AAPS PharmSciTech*.

[15] Young J. F. (1972). A review of the mechanisms of set-retardation in portland cement pastes containing organic admixtures. *Cement and Concrete Research*.

[16] Pourchez J., Grosseau P., Ruot B. (2010). Changes in C3S hydration in the presence of cellulose ethers. *Cement and Concrete Research*.

[17] Ou Z. H., Ma B. G., Jian S. W. (2012). Influence of cellulose ethers molecular parameters on hydration kinetics of Portland cement at early ages. *Construction and Building Materials*.

